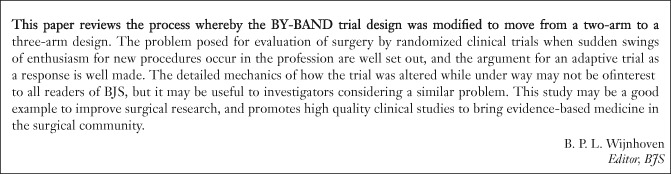# Adaptation of the By‐Band randomized clinical trial to By‐Band‐Sleeve to include a new intervention and maintain relevance of the study to practice

**DOI:** 10.1002/bjs.10562

**Published:** 2017-07-13

**Authors:** C. A. Rogers, B. C. Reeves, J. Byrne, J. L. Donovan, G. Mazza, S. Paramasivan, R. C. Andrews, S. Wordsworth, J. Thompson, J. M. Blazeby, R. Welbourn, the By‐Band‐Sleeve study investigators, S. Agrawal, S. Ajaz, Y. Koak, A. Ahmed, N. Fakih, S. Hakky, K. Moorthy, S. Purkayastha, S. Awad, K. Fareed, P. Leeder, S. Balupuri, W. Carr, N. Jennings, P. Small, R. Byrom, N. Davies, N. Carter, B. Knight, S. Somers, V. Charalampakis, M. Daskalakis, R. Nijar, M. Richardson, R. Singhal, P. Super, M. Clarke, A. Cota, I. Finlay, S. Dexter, J. Hayden, S. Mehta, A. Sarela, J. Kelly, D. Mahon, H. Noble

**Affiliations:** ^1^ Clinical Trials and Evaluation Unit, School of Clinical Sciences University of Bristol Bristol UK; ^2^ Centre for Surgical Research, School of Social and Community Medicine University of Bristol Bristol UK; ^3^ Department of General Surgery University Hospital Southampton NHS Foundation Trust Southampton UK; ^4^ Department of Diabetes and Endocrinology Musgrove Park Hospital, Taunton and Somerset NHS Trust Taunton UK; ^5^ Department of Upper Gastrointestinal and Bariatric Surgery Musgrove Park Hospital, Taunton and Somerset NHS Trust Taunton UK; ^6^ University of Exeter Medical School Exeter UK; ^7^ Health Economic Research Centre University of Oxford Oxford UK; ^8^ School of Sport and Exercise Sciences University of Birmingham Birmingham UK; ^9^ Homerton University Hospital London; ^10^ Imperial College Healthcare NHS Trust London; ^11^ Royal Derby Hospital Derby; ^12^ Sunderland Royal Hospital Sunderland; ^13^ Royal Bournemouth and Christchurch Hospitals; ^14^ Queen Alexandra Hospital Portsmouth; ^15^ Heart of England NHS Foundation Trust Birmingham; ^16^ Royal Cornwall Hospital Truro; ^17^ St James's University Hospital Leeds; ^18^ University Hospital Southampton Southampton; ^19^ Musgrove Park Hospital Taunton

## Abstract

**Background:**

Recruitment into surgical RCTs can be threatened if new interventions available outside the trial compete with those being evaluated. Adapting the trial to include the new intervention may overcome this issue, yet this is not often done in surgery. This paper describes the challenges, rationale and methods for adapting an RCT to include a new intervention.

**Methods:**

The By‐Band study was designed in the UK in 2009–2010 to compare the effectiveness of laparoscopic adjustable gastric band and Roux‐en‐Y gastric bypass for severe obesity. It contained a pilot phase to establish whether recruitment was possible, and the grant proposal specified that an adaptation to include sleeve gastrectomy would be considered if practice changed and recruitment was successful. Information on changing obesity surgery practice, updated evidence and expert opinion about trial design were used to inform the adaptation.

**Results:**

The pilot phase recruited over 13 months in 2013–2014 and randomized 80 patients (79 anticipated). During this time, major changes in obesity practice in the UK were observed, with gastric band reducing from 32·6 to 15·8 per cent and sleeve gastrectomy increasing from 9·0 to 28·1 per cent. The evidence base had not changed markedly. The British Obesity and Metabolic Surgery Society and study oversight committees supported an adaptation to include sleeve gastrectomy, and a proposal to do so was approved by the funder.

**Conclusion:**

Adaptation of a two‐group surgical RCT can allow evaluation of a third procedure and maintain relevance of the RCT to practice. It also optimizes the use of existing trial infrastructure to answer an additional important research question. Registration number: ISRCTN00786323 (http://www.isrctn.com/).

## Abstract 2

Something to consider

## Introduction

Large‐scale RCTs in surgery can be difficult to design and conduct. There are challenges with recruitment, intervention complexity and outcome assessment. Recruitment may be slow because surgeons and associated clinical teams (such as anaesthetists, nurses, dieticians and psychologists) are unfamiliar with presenting uncertainty and recruiting patients, and there is often a lack of clinical trials research infrastructure in surgical departments^1^, ^2^, ^3^. As a result, extensions to the recruitment period may be required or the trial may be stopped early before the target sample size is reached^4^, ^5^. The additional time taken to deliver surgical trials has important implications over and above increased research costs, meaning that the initial research question can become outdated owing to changing practice. Often, new interventions are introduced while the trial is recruiting, despite a lack of evidence from RCTs for effectiveness. This may be due to ‘fashion and popular opinion’, ‘common sense/plausibility’, ‘marketing by industry’ or by ‘influential opinion leaders’. Surgical practice can therefore change, and new interventions can be widely implemented, without evaluation and before outcomes from the trial are available. This contrasts with the introduction of new medicines, which requires formal evaluation and strict adherence to governance procedures before licensing for general use.

The adoption of new but unevaluated surgical interventions outside a trial can threaten the viability of an ongoing trial in a number of ways. Participating surgeons may want to undertake the new intervention, reducing the proportion of patients offered the trial. In the case of a new medication, this can be controlled to some extent by a commissioning decision that the medication should be available ‘only in research’^6^. Although this option may be available for surgical interventions in some countries, surgeons are rarely willing to apply it voluntarily. Thus, patients can remain outside a trial and have the new intervention, as is evidenced by the way in which new surgical interventions are often introduced and adopted^7^, ^8^. In the absence of an ‘only in research’ ruling, patients themselves may prefer the new intervention (albeit based on limited evidence and understanding). It is also possible that centres and surgeons may choose not to join the trial because the research question may appear obsolete, although there is a lack of high‐quality evidence to inform such judgements.

One approach to keeping a trial relevant is to use an adaptive design. The term is used most often in the context of changing interventions, or respecifying the comparator, as evidence emerges about the interventions being evaluated^9^. For example, by evaluating short‐term outcomes (or side‐effects), a decision can made to drop an intervention when the accruing data confidently show that it could never be effective – on grounds of futility. Importantly, adaptive trial protocols specify criteria for adding or dropping interventions before the trial begins. Although there is increasing knowledge and acceptance of the use of adaptive trial designs for novel pharmaceutical interventions, the principle has been applied less often in trials of surgery.

Adapting a surgical trial is challenging. It may be difficult to agree when a new surgical intervention is sufficiently well developed and standardized for evaluation, and to establish when surgeons have sufficient experience of it to contribute to its evaluation in a trial.

The aim of this article is to describe methods that were used to adapt a surgical RCT to include an additional, emerging surgical technique. The methods were applied in what started as the By‐Band study of two different bariatric surgical techniques^10^, now adapted to the By‐Band‐Sleeve study to include an additional evaluation of sleeve gastrectomy.

## Methods

The By‐Band study was a multicentre pragmatic RCT undertaken in the UK with the aim to evaluate the effectiveness and cost‐effectiveness of two interventions, laparoscopic adjustable gastric band and laparoscopic Roux‐en‐Y gastric bypass. It was designed in two phases. The first phase was set up in two centres to examine recruitment. This internal pilot phase included formal progression criteria/goals (*Table* 1), which were prespecified and approved by the funder. Formal review of phase 1 was scheduled to take place after the study had been open for 2 years. The funder agreed that if the criteria/goals were met the trial could continue into the second phase. Full details of the protocol are available elsewhere^10^. The protocol allows all eligible trial participants to join the study. Those who do not consent to randomization are invited to contribute data on the operation chosen and participate in follow‐up. These data will be used to understand the generalizabilty of the study results.

**Table 1:** Details of pre‐agreed recruitment and retention goals (‘progression criteria’) in the internal pilot phase of the study, and actual progress achieved

Pre‐agreed goals to be achieved	Actual progress achieved
To screen 400 patients	333 patients were screened (83 per cent of that expected in original grant application)
Sixty per cent of screened patients were eligible	231 patients (69·4 per cent) were found to be eligible
To increase recruitment rates from 30 per cent over the first 18 months of recruitment, rising to 50 per cent thereafter*	Recruitment rates were 27 per cent over the first 6 months of recruitment, rising to 39 per cent thereafter (target 79 randomized, achieved 80)
Less than 5 per cent did not receive allocated treatment	2 of 57 (4 per cent) failed to receive the allocated treatment
Less than 5 per cent lost to follow‐up	1 (2 per cent) withdrawn
To reconsider the role of sleeve gastrectomy and whether a three‐group study should be proposed	Sleeve data presented. The proposal to adapt the trial was approved by the funder

* Recruitment rate is the percentage of eligible patients consenting to join the randomized study.

When the study was designed in 2009–2010, gastric band and bypass accounted for 80 per cent of all bariatric operations in the National Health Service (NHS)^11^. A new operation, sleeve gastrectomy, was starting to be performed, but accounted for only 8 per cent of operations. Surgeons' experience of sleeve gastrectomy was limited, few long‐term outcome data were available, and there was no consensus on how the operation should be performed. The By‐Band research team therefore decided that it was not appropriate to include sleeve gastrectomy as a third group at that time. This information was presented in the grant application, with a brief proposal to review with the trial steering committee the case for adding a sleeve group at the end of the internal pilot phase. The proposal recognized that, if the trial was adapted, an increase in sample size would likely be needed.

### Criteria for progression from the pilot to the main trial

The goals used for progression from the phase 1 pilot to the main trial (phase 2) comprised five recruitment and protocol adherence‐based measures. The study team also planned to develop a core outcome set during the pilot (*Table* 1)^12^. An intervention to optimize recruitment was included in the pilot because of well recognized strong preferences for interventions amongst patients and surgeons. The Quintet Recruitment Intervention (QRI) was used^13^. This has two major components: understanding recruitment as it happens and then developing a plan of action to address identified difficulties; and optimizing informed consent in collaboration with the RCT chief investigator and the clinical trials unit. The QRI has been used successfully in several surgical trials.

### Methods for proposing an adaptation to include sleeve gastrectomy

Four sources of information were reviewed to determine whether adaptation to include sleeve gastrectomy should be recommended: data on current surgical practice including both NHS and private healthcare provision; published comparative evidence and ongoing trials; expert surgical opinion (trial steering and data monitoring and safety committees, and specialist society); and published data on the stability of sleeve gastrectomy. The evidence collated was submitted to the funder with the proposal to adapt the trial to include a third group, changing the trial identity to By‐Band‐Sleeve.

### Implementation of the adaptation

A list of practical and logistical steps needed to implement an adaptation from two to three groups was drawn up. These steps included considering the implications for trial participants at different stages of the trial (for example, invited to take part but not consented, consented but not randomized, randomized but awaiting surgery, in follow‐up). A strategy for how to proceed was prepared.

## Results

### Progression from phase 1 to phase 2 of the trial

The grant opened in January 2012 and the formal review of phase 1 took place in January 2014. The study opened to recruitment in the first centre in November 2012, and in the second in February 2013. Between November 2012 and December 2013, 80 patients were recruited and randomized (79 anticipated). The other goals for progression to the main trial were also met (*Table* 1). The funder therefore agreed that the trial should progress to phase 2 and expand. Eleven centres are currently participating in the trial; four phase 2 centres opened before the adaptation to By‐Band‐Sleeve, and five opened after the trial had been adapted.

### Adaptation to include sleeve gastrectomy

#### Changes in current surgical practice

Data from the National Bariatric Surgery Registry were reviewed to inform the rates of each type of operation^11^. Rates of sleeve gastrectomy increased from 9·0 to 28·1 per cent during phase 1 (*Table* 2). Over the same period, the rates for gastric bypass remained stable and the proportion of operations using gastric band surgery declined from 32·6 to 15·8 per cent overall. Analysis of data by NHS and private provision showed that, in the NHS, gastric band surgery accounted for just 11·0 per cent of bariatric procedures in 2013, but in the private health sectors band surgery remained the most common operation (*Table* 2). This pattern was mirrored elsewhere in the world^14^.

**Table 2:** Rates of laparoscopic Roux‐en‐Y gastric bypass, laparoscopic adjustable gastric band and sleeve gastrectomy in the National Health Service and private sector, 2008–2013

	2008–2009	2011	2012	2013
NHS and private practice				
Band	2132 (32·6)	1316 (25·5)	1358 (24·8)	891 (15·8)
Bypass	3817 (58·4)	3030 (58·8)	2894 (52·9)	3176 (56·2)
Sleeve	588 (9·0)	809 (15·7)	1218 (22·3)	1587 (28·1)
Total	6537	5155	5470	5654
NHS only (HES data)				
Band	n.a.	637 (16·9)	736 (18·2)	506 (11·0)
Bypass	n.a.	2540 (67·6)	2394 (59·1)	2816 (61·1)
Sleeve	n.a.	583 (15·5)	922 (22·8)	1290 (28·0)
Total	n.a.	3760	4052	4612
Private practice only*				
Band	n.a.	679 (48·7)	622 (43·9)	385 (36·9)
Bypass	n.a.	490 (35·1)	500 (35·3)	360 (34·5)
Sleeve	n.a.	226 (16·2)	296 (20·9)	297 (28·5)
Total	n.a.	1395	1418	1042

Values in parentheses are percentages.

* Data likely to be under reported. NHS, National Health Service; HES, Hospital Episodes Statistics; n.a., not available.

#### Published comparative evidence and ongoing trials

The Cochrane systematic review of bariatric surgery that had informed the original By‐Band grant application was updated and published in 2014^15^. The authors concluded that there remained a lack of high‐quality evidence for the different types of bariatric surgery. The trials reviewed were primarily small single‐centre studies performed outside the UK. They were at risk of bias and focused predominantly on short‐term outcomes up to 1 year. Interrogation of clinical trials registries identified three ongoing trials evaluating sleeve gastrectomy^16^, ^17^, ^18^. These were also small, non‐UK, single‐centre studies. The evidence from completed trials, and the evidence that would be generated from ongoing trials, was judged to be inadequate to draw any definitive conclusion about the effectiveness of the three types of operation.

#### Expert opinion

The proposal to adapt the trial was discussed at a special session of the British Obesity and Metabolic Surgery Society annual meeting in January 2014. Those present supported it, and additional centres were recruited. The independent trial steering and data monitoring committees supported the adaptation.

#### Intervention stability

At the time when the trial was first designed there was little consensus regarding sleeve gastrectomy itself and how it should be performed and followed up. By the time of the review, best practice guidelines had been published and the fourth international consensus summit for sleeve gastrectomy had taken place^19^, ^20^. Meetings confirmed that the procedure was safe, and agreed standards for its conduct were documented.

#### Research question

The proposal for adaptation to a three‐group trial was reviewed and accepted by the funder. The original research question, (1) below, was expanded in the three‐group trial to answer three questions based on co‐primary outcomes:

1. Does gastric bypass lead to better quality of life and at least as good weight loss as gastric band (original question)?
2. Does sleeve gastrectomy lead to better quality of life and at least as good weight loss as gastric band (new question)?
3. Does sleeve gastrectomy lead to better quality of life and at least as good weight loss as gastric bypass (new question)?

**Figure 1:**
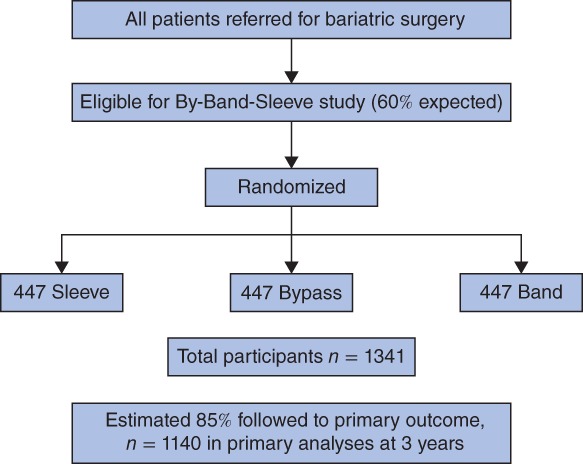
By‐Band‐Sleeve trial design

When calculating the sample size for the adapted design, the same assumptions as for the original calculations were used^10^. Because the number of hypotheses was increased threefold, the significance levels were adjusted from 5 to 2 per cent for the two‐sided statistical tests of superiority and from 2·5 to 1 per cent for the one‐sided statistical tests of non‐inferiority. Under these assumptions, the revised total sample size for a trial with equal allocation across the three treatment groups was 1341 (447 per group) (*Fig*. 1).

The formal proposal for the adaptation was submitted at the same time as the request to progress from the pilot to the main trial. Approval and additional funding to support the adaptation was granted in October 2014.

### Implementation of the adaptation

Practical and logistical changes necessary to adapt a surgical trial are outlined in Table 3. All patient‐facing study documents had to be revised and submitted as a major amendment to the research ethics committee for approval. Data collection forms needed to be extended to capture the additional surgical procedure. The randomization scheme had to be revised to allow future participants to be allocated to one of the three surgical procedures. The allocation ratio was modified to ensure there would be approximately equal numbers of participants per group at the end of the trial, to provide the same power for all comparisons. Adjustment to the allocation ratio was determined by simulation to ensure balance was achieved, while at the same time ensuring that allocation would be unpredictable to prevent allocation bias. The allocation ratio chosen was specific to each study centre, depending on the numbers recruited before the adaptation and the projected recruitment rate going forward. Each centre needed guidance on: when to collect data using the By‐Band data collection forms and when to collect data using the revised By‐Band‐Sleeve forms; when to start using the revised patient information leaflets; and when the randomization system and database would be switched over and how to manage participants who had given consent but had not been randomized at the time of the switch (Table 3).

**Table 3:** Practical and logistical considerations for optimizing adaptation of a two‐group to three‐group surgical trial

Consideration	Potential problems	Suggestions to optimize adaptation
Study logo and acronym	If the acronym and study logo are specific to the two groups (e.g. By‐Band), they will need to be changed (e.g. to By‐Band‐Sleeve). This may: (1) create changes to study and website materials that would not otherwise be necessary, and (2) jeopardize the branding of the study	At the outset, select an acronym and study logo that could encompass a future adaptation
Data collection forms	New and updated case report forms needed. This may: (1) take a lot of time and involve significant changes to the forms, and (2) lead to major changes to the database	Design case report forms in logical sections. For example, separate information and adverse event data that are common for all surgical procedures from that which is specific to one procedure. Organizing the data collection in this way can help minimize the database changes needed
Allocation of procedures	If equal allocation of participants to groups is applied after the adaptation, this will result in unbalanced numbers of participants in each group at the end of the trial	Close working with senior statisticians is recommended. If the allocation ratio is adjusted, simulation of future recruitment at each centre is needed to ensure that the allocation remains concealed and cannot be predicted. Rigorous testing of all changes needs to be performed
	Alternatively, the allocation ratio can be adjusted so that the numbers per group are approximately equal at the end of the trial when the target sample size is reached. In modifying the allocation ratio, it is necessary: (1) to consider the projected recruitment rate and numbers recruited already in each centre before the adaptation, and (2) to assess the impact the change of ratio will have on future recruitment	
		This work would need to be included in the overall cost of the adaptation
Transition pathway for participants at different stages of trial	Information provision for participants during trial adaption needs consideration, especially for those part way through recruitment. Each (potential) participant will be at one of five stages; they may have: (1) been sent information about the two‐group study but not yet had a consultation, (2) discussed the two‐group study but not yet consented to randomization, (3) been consented to the two‐group study but not yet randomized, (4) been randomized and awaiting surgery, or (5) undergone surgery and be in follow‐up	It is recommended that the trial team discusses the transition with individual centres. It is particularly important to agree the process for patients at stages (1) and (3). In By‐Band‐Sleeve, these patients had had a consultation in which two procedures were discussed and it was not feasible for them to have a further consultation. Randomization for these patients was therefore handled centrally by the trials unit to prevent them being allocated to the new procedure

**Figure 2:**
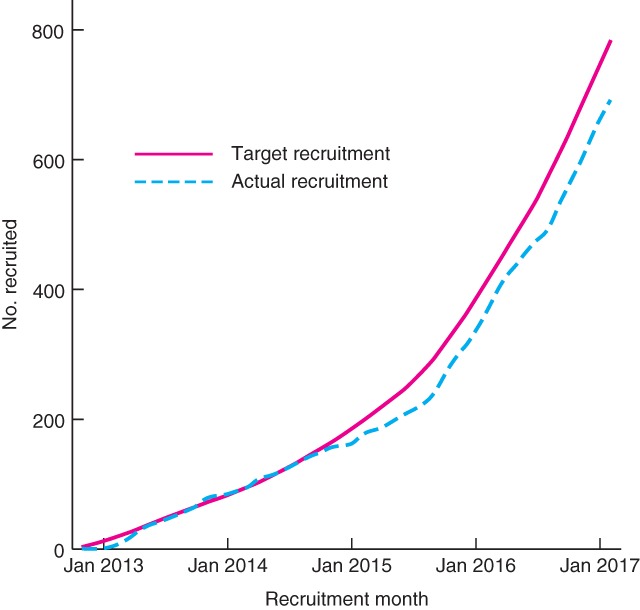
Recruitment into the By‐Band‐Sleeve study at January 2017

The adapted By‐Band‐Sleeve study opened to recruitment in August 2015, 13 months after approval from the funder to proceed with the adaptation. The transition went smoothly and the study is recruiting steadily to all three procedures (Fig. 2). In February 2017, the 50 per cent recruitment milestone was reached (671 patients randomized into the trial). All study centres continue to be supported with the QRI^13^.

## Discussion

This paper describes adaptation of a two‐group RCT in bariatric surgery to a three‐group trial. The adaptation was proposed because of changing clinical practice, namely the increasing uptake of a new procedure in the absence of high‐quality evidence of its clinical effectiveness compared with the procedures already being evaluated. The trial team was aware of the interest in the new procedure when designing the By‐Band study, and specified the possibility of an adaptation in the original grant proposal. This stated that if recruitment in the internal pilot phase proved possible the trial team would consider whether adapting to a three‐group trial was relevant (and present a new proposal to the funder). This approach to designing and conducting RCTs in surgery is recommended, although not often used. It ensures the continued relevance of the trial to surgeons, allows efficient use of trial resources, and enables a procedure that has stabilized and is being widely used to undergo comparative rigorous evaluation.

Adapting a surgical RCT has proved challenging, with practical and scientific implications and complexities. The majority of experience has been gained in pharmaceutical studies where the pressure to adapt a trial arises from the emergence of competing ‘like‐me’ drugs^9^, and with pressure to evaluate multiple new drugs. In pharmaceutical trials short‐term or surrogate endpoints are typically used to inform adaptation decisions. In trials with longer‐term endpoints (the primary endpoint of By‐Band‐Sleeve is at 3 years), it is not sensible to adapt the trial based on short‐term outcomes, especially if no appropriate short‐term surrogate measure exists. However, as shown here, there is an opportunity to introduce additional group(s) to evaluate new intervention(s). The By‐Band‐Sleeve study also includes a ‘non‐randomized’ cohort of eligible patients who consent to data collection and follow‐up but not to randomization. Analysis of this cohort will provide important insights into how the trial has been integrated into each clinical practice at the study centres.

One challenge to overcome when a study changes from two to three groups is to ensure that trial processes are not disrupted during the transition and that patients continue to be recruited and followed up in accordance with the protocol. For example, some patients were consented to a two‐group trial but, by the time they were randomized, the trial had switched to three groups. It was not feasible to have a further consultation to discuss the trial again, so the randomization for these patients was handled centrally by the trials unit to avoid them being allocated to the new procedure.

Another decision that needs to be made is whether the allocation ratio should be chosen to create approximately equally sized groups at the end of the trial, or whether the ratio should be 1 : 1 : 1 going forward, accepting that the sample size for the two original study groups will be larger than that for the new group. In the By‐Band‐Sleeve adaptation, the allocation ratio at each centre was adjusted to achieve approximately equal allocation overall. Although the revised allocation ratio should provide comparable power for all group comparisons, it is not without ‘risks’. A balanced allocation may not be achieved if the trial stops early for any reason, or if the recruitment rates differ significantly from those predicted.

The primary reason for adapting a trial to include a new procedure is to keep it up‐to‐date and relevant to clinical practice. It is, however, important to adapt a trial and include a new procedure only when surgeons are comfortable to discuss clinical equipoise with patients. These issues are challenging, because surgeons may naturally believe that the new procedure has advantages and they will find it more interesting/exciting to do a new procedure rather than a standard technique. In these situations, surgeons may state that a randomized evaluation is not possible because it is ‘too late’ and they do not have clinical equipoise. This view is represented in Buxton's law which says: ‘it is always too early for rigorous evaluation until, unfortunately, it's suddenly too late’^21^. Despite this established mantra, there have been several recent examples of successful surgical trials in which surgeons have been supported to provide balanced information to inform patient decision‐making and ensure successful recruitment^22^, ^23^. Because of the success of these other trials, this support was included in By‐Band and By‐Band‐Sleeve. The QRI uses qualitative methods to understand how surgeons communicate information to patients. Consultations are audiorecorded and analysed, and meetings are held with surgeons to provide training in how to optimize informed consent. Although this approach to supporting trial recruitment represents progress for randomized trials, there is a need to use these methods to optimize information provision in other settings when surgeons talk to patients, such as during early‐phase studies and with the introduction of novel techniques.

This article describes an example of adaption from a two‐ to a three‐group surgical trial to include an increasingly prevalent intervention and allow its evaluation in a pragmatic study. The inclusion of an internal pilot and a formal review of success in recruiting to the two‐group design provided a convenient interval in which to consider whether a third group should be added. The justification was based primarily on external sources of information, in contrast to trial adaptation based on accruing evidence within the trial. This approach is recommended for RCTs in surgery to optimize the efficiency of the trial team infrastructure, allow surgeons to undertake an additional intervention, and maintain the relevance of the research question.

## Collaborators

Other By‐Band‐Sleeve study investigators (principal investigator denoted by an asterisk): S. Agrawal*, S. Ajaz, Y. Koak (Homerton University Hospital, London); A. Ahmed*, N. Fakih, S. Hakky, K. Moorthy, S. Purkayastha (Imperial College Healthcare NHS Trust, London); S. Awad, K. Fareed, P. Leeder* (Royal Derby Hospital, Derby); S. Balupuri, W. Carr, N. Jennings*, P. Small (Sunderland Royal Hospital, Sunderland); R. Byrom, N. Davies* (Royal Bournemouth and Christchurch Hospitals); N. Carter*, B. Knight, S. Somers (Queen Alexandra Hospital, Portsmouth); V. Charalampakis, M. Daskalakis, R. Nijar, M. Richardson, R. Singhal*, P. Super (Heart of England NHS Foundation Trust, Birmingham); M. Clarke, A. Cota, I. Finlay* (Royal Cornwall Hospital, Truro); S. Dexter, J. Hayden*, S. Mehta, A. Sarela (St James's University Hospital, Leeds); J. Kelly (University Hospital Southampton, Southampton); D. Mahon, H. Noble (Musgrove Park Hospital, Taunton).

## Editor's comments